# Grand Challenges in Fungal Biotechnology

**DOI:** 10.3389/ffunb.2020.626551

**Published:** 2020-12-08

**Authors:** Scott E. Baker

**Affiliations:** ^1^Joint BioEnergy Institute, Emeryville, CA, United States; ^2^Functional and Systems Biology Group, Environmental Molecular Sciences Division, Pacific Northwest National Laboratory, Richland, WA, United States

**Keywords:** fungi, biotechnology, genome, synthetic biology, genetics, metabolites, enzymes, secretion

Intertwined for millennia with the human experience, fungi perform vital functions in our daily life. Without fungi we would not have bread or beer, sake or soy sauce, tofu or miso. Fungi secrete enzymes that deconstruct the world around them into nutrient building blocks which they then absorb for nutrition. For example, the number of enzymes for carbohydrate assimilation continues to expand (Garron and Henrissat, 2019). In addition, fungi communicate and manipulate their environment by secreting a stunning variety of metabolites, especially secondary metabolites (Keller et al., 2005; Keller, 2019). Thus, the ability of fungi to produce and secrete prodigious amounts of proteins and metabolites is central to their value in biotechnology.

Below I outline a selection of current challenges in fungal biotechnology. The list of challenges and the examples cited are not meant to be exhaustive.

## Metabolic Pathways

Fungi are a rich and diverse source of metabolic pathways. Through history there are examples of fungal production hosts for several different types of metabolites. One of the early fungal metabolites to be produced on an industrial scale was citric acid. The industrialization of fungal organic acid production arguably began when USDA researcher James Currie published and patented the *Aspergillus niger* citric acid process in the US (Currie, 1917). By the late 1920s, *A. niger* had displaced lemons as the source of citric acid (Neushul, 1993; Lombardino, 2000). Over 100 years after the process was patented and published, we are only now identifying the genes responsible for regulation and export of citric acid (Niu et al., 2015; Steiger et al., 2019). Industrial production of organic acids is important for food and chemical industry with many research challenges that must be addressed to enable a robust bioeconomy.

Another success for fungal metabolite production occurred during the World War II when teams of researchers from academia, government and industry discovered new fungal strains and developed processes leading to greatly increased production of penicillin, which had been discovered in 1928 by Alexander Fleming (Fleming, 1929; Neushul, 1993; Lombardino, 2000). Phylogenetic and genomic analyses have been used to definitively identify the species of Fleming's isolate as well as show that one of the critical steps in development of strains with increased penicillin production is gene duplication of the biosynthetic gene cluster (van den Berg et al., 2008; Houbraken et al., 2011; Pathak et al., 2020). As new fungal natural products are discovered, it will be critical to understand the biochemistry and regulation of their associated biosynthetic clusters for optimal compound characterization and production host development.

Food and beverage production that involves fungi is also a platform for biotechnological innovation. *Aspergillus oryzae*, a koji fungus, was among the first filamentous fungal genomes to be sequenced; this achievement laid the foundation for genome scale based insights into domestication of fungi for these processes (Machida et al., 2005, 2008; Kjaerbolling et al., 2020). Used in bread, wine, sake and beer production, genomic studies of *Saccharomyces cerevisiae* are shedding light on domestication and specialization within this species (Gallone et al., 2016). With an arsenal of tools available for molecular genetic manipulation, *S. cerevisiae* has been modified to produce the terpene involved in the hoppy flavor so prevalent in IPA style beer (Denby et al., 2018). Continuing to improve our understanding of fungal biology as it relates to food and beverage production will lead to improved processes and production efficiency.

## Protein Secretion

Not only are they able to derive nutrition from a broad range of building block molecules, fungi secrete a massive array of enzymes to digest complex substrates into building block nutrient compounds. As such, fungi have long been utilized as hosts for protein production. Indeed, recognizing the enzyme production potential of fungi, Jokichi Takamine was granted one of the first biotechnology patents in 1894 for development of a fungal derived digestive enzyme mixture (Takamine, 1894).

Genome sequencing and molecular analysis continues to provide insight into how fungi regulate their secretome in response to different types of biomass (Benocci et al., 2017; Wu et al., 2020). As the molecular machinery responsible for protein secretion continues to be uncovered, there is increased opportunity for rewiring and improving production hosts (Baker, 2018). While fungi are known to secrete high amounts of digestive enzymes, regulation of their secretion is tightly controlled and despite significant progress, unraveling the regulatory circuits controlling fungal nutrient acquisition remains a massive challenge.

## Synthetic and Systems Biology

Once primarily the domain of model organisms, genetic tractability has been democratized by CRISPR-cas9 methods (Satish et al., 2020). An attractive target for future genetic engineering method development includes anaerobic fungi who produce scaffolded plant biomass degrading enzyme complexes called cellulosomes (Hooker et al., 2019; Wilken et al., 2020). The potential to manipulate single genes, families of related genes or even the whole genome is now possible using CRISPR-cas9 based approaches (Schwartz et al., 2019). Using whole genome genetic queries to screen for genes whose products are involved in controlling morphology and control protein and metabolite secretion has potential to accelerate the development of industrial fungal production hosts.

The chemical diversity present in the fungal kingdom makes fungi attractive synthetic biology chassis. Understanding the dynamic nature of metabolic pathways and cell signaling is essential for both understanding and designing biosystems. The development of metabolic and regulatory models for a variety of fungi is made tractable by the rapid generation of new genome sequences and genome scale data (Swift et al., 2019). Combining genome scale modeling with novel genetic tools make fungi increasingly attractive as cell factories for small molecule production (Lim et al., 2012; Unkles et al., 2014; Schwartz et al., 2019).

## Conclusion

The last century has seen great advances in fungal biotechnology from production of enzymes and organic acids to genome sequencing, engineering and modeling. Despite these advances there are still many aspects of fungal biology with potential applications in biotechnology we still do not fully understand. The future will see research focused on translating genomes into functional and phenotypic information that will inform design of fungal biotechnology based solutions to grand challenges in human health, nutrition, sustainable energy, and the environment.

## Author Contributions

SEB conceived and wrote the manuscript.

## Conflict of Interest

The author declares that the research was conducted in the absence of any commercial or financial relationships that could be construed as a potential conflict of interest.
